# A software tool ‘CroCo’ detects pervasive cross-species contamination in next generation sequencing data

**DOI:** 10.1186/s12915-018-0486-7

**Published:** 2018-03-05

**Authors:** Paul Simion, Khalid Belkhir, Clémentine François, Julien Veyssier, Jochen C. Rink, Michaël Manuel, Hervé Philippe, Maximilian J. Telford

**Affiliations:** 1 Institut des Sciences de l’Evolution (ISEM), UMR 5554, CNRS, IRD, EPHE, Université de Montpellier, Montpellier, France; 2 Max Plank Institute of Molecular Cell Biology and Genetics, Pfotenhauerstrasse 108, 01307 Dresden, Germany; 3 Sorbonne Université, CNRS, Institut de Biologie Paris-Seine (IBPS), Evolution Paris-Seine (UMR7138), Case 05, 7 Quai St Bernard, 75005 Paris, France; 4 Centre de Théorisation et de Modélisation de la Biodiversité, Station d’Ecologie Théorique et Expérimentale, UMR CNRS 5321, Moulis, 09200 France; 5 Département de Biochimie, Centre Robert-Cedergren, Université de Montréal, Montréal, H3C 3J7 Québec Canada; 6 Centre for Life’s Origins and Evolution, Department of Genetics Evolution and Environment, University College London, Darwin Building, Gower Street, London, WC1E 6BT UK

**Keywords:** Contamination, NGS, Phylogenomics, Ctenophora

## Abstract

**Background:**

Multiple RNA samples are frequently processed together and often mixed before multiplex sequencing in the same sequencing run. While different samples can be separated post sequencing using sample barcodes, the possibility of cross contamination between biological samples from different species that have been processed or sequenced in parallel has the potential to be extremely deleterious for downstream analyses.

**Results:**

We present CroCo, a software package for identifying and removing such cross contaminants from assembled transcriptomes. Using multiple, recently published sequence datasets, we show that cross contamination is consistently present at varying levels in real data. Using real and simulated data, we demonstrate that CroCo detects contaminants efficiently and correctly. Using a real example from a molecular phylogenetic dataset, we show that contaminants, if not eliminated, can have a decisive, deleterious impact on downstream comparative analyses.

**Conclusions:**

Cross contamination is pervasive in new and published datasets and, if undetected, can have serious deleterious effects on downstream analyses. CroCo is a database-independent, multi-platform tool, designed for ease of use, that efficiently and accurately detects and removes cross contamination in assembled transcriptomes to avoid these problems. We suggest that the use of CroCo should become a standard cleaning step when processing multiple samples for transcriptome sequencing.

**Electronic supplementary material:**

The online version of this article (10.1186/s12915-018-0486-7) contains supplementary material, which is available to authorized users.

## Background

Contamination between nucleic acid samples has long been recognised as a potential problem in molecular biology. The use of amplification by polymerase chain reaction (PCR) and, more recently, high-throughput sequencing, implies that even very low levels of contaminating nucleic acids, regardless of their sources, can be sequenced at sufficient coverage to be present in downstream datasets [1–9]. Various tools have already been developed in order to discriminate between the sequences of the organism of interest and contaminant sequences originating from parasites, gut bacteria, endosymbionts or the environment. These algorithms usually identify the contaminant sequences based on specific criteria and infer the taxonomic source of the contaminant using a reference database. The Blobtools pipeline [10] detects contaminant sequences based on their GC content, read coverage and taxonomic assignment (using Basic Local Alignment Search Tool (BLAST) against the National Center for Biotechnology Information (NCBI) non-redundant database). A slightly different method, Anvi’o [11], first automatically bins contigs based on read coverage and/or k-mer frequencies, then identifies the contaminant bins. Lastly, the algorithm Model-based Categorical Sequence Clustering (MCSC) [5] uses a clustering method based on the frequent patterns observed in the sequences (divisive hierarchical clustering) and then identifies the contaminant clusters by blasting against the UniRef90 database. These methods (except for MCSC) focus on genomic data; however, they partially rely on public databases that are not always devoid of contamination and are designed to detect contamination from distant organisms. As transcriptomic data are currently widely used in evolutionary biology, we designed a new tool, CroCo, designed for RNA sequencing (RNA-seq) data; it relies on expression level estimates, it is reference-free, and it targets another type of contamination: cross contamination.

Cross contamination is defined as contamination across samples handled in parallel in a given sequencing project. It is of experimental origin and can potentially arise at multiple benchwork steps: sample handling, DNA/RNA extraction, library preparation and amplification, sample multiplexing and inaccurate barcode sequencing. Our empirical observations show that some amount of cross contamination seems unavoidable when multiplexing complementary DNA (cDNA) libraries for high-throughput transcriptome sequencing of multiple species (for instance, for subsequent phylogenetic tree reconstruction). The phenomenon is obvious when finding sequences that are identical or almost identical at the nucleotide level in assembled transcriptomes of two or more sufficiently distantly related species (for an example, see Additional file 1: Figure S1). Such cases have already been detected in several recent evolutionary biology studies [9, 12–16]. Cross contamination creates false similarities between species, with obvious deleterious consequences for any kind of downstream comparative analysis.

## Implementation

### Methods

In order to determine whether a given sequence in a sequencing experiment involving multiple species is likely to be a contaminant, we have developed a procedure that uses transcript quantification estimates. Our approach is independent from any public database. In short, a sequence present in the assembled transcriptome of species A will be considered a contaminant if the same sequence is represented by a higher number of reads in another species B from the same study. This procedure relies on the following assumptions: (1) contamination is likely to derive from messenger RNAs (mRNAs) that are at high concentration in the source species (both because these are more likely to contaminate and because successfully assembling a transcript requires sufficient read coverage); (2) contaminating molecules are expected to be found in lower quantities in the contaminated sample than in their sample of origin; (3) the ratio between levels of contaminator and contaminated sample should hold regardless of the origin of cross contamination (e.g. tissue/RNA contamination at the bench, mixed tag during double PCR, contaminations during manufacturer’s proprietary protocols), the determination of which is not the focus here (but see [9]). It is therefore expected that reads from contaminating nucleic acids will be found at much lower levels in the contaminated sample than in the contaminating source.

Here we present CroCo, a cross contamination detection and removal tool based on these expectations, which expands on preliminary pipelines used in two independent recent studies [15, 16]. CroCo uses sequence read files and assembled transcriptomes from each sample included in a given sequencing project involving multiple species. First, a BLASTN step across all pairwise transcriptomes defines a list of transcripts suspiciously similar (at the nucleotide level) across samples. By default, two transcripts in two different samples that are > 95% similar over a fragment of > 40 nucleotides are considered suspicious. This criterion is valid as long as the genetic distances between all species are sufficiently high (see below). All assembled transcriptome files are then concatenated into a reference metatranscriptome for subsequent estimations of their expression levels. For a given suspicious transcript, CroCo quantifies its expression level *N* (in transcripts per million, TPM — see [17]) in reads from each sample. A transcript present in the assembly from sample A but expressed in another sample at a higher level (that passes a user-defined threshold) is considered a contaminant. CroCo performs this comparison for all suspicious transcripts from all samples. Given three user-defined parameters corresponding to (1) a fold difference value (*X*, default = 2); (2) a high expression level (*Y*, default = 300 TPM); and (3) a low expression level (*Z*, default = 0.2 TPM), and where *Nf* is the expression level in the focal sample and *Na* is the expression level in alien samples, it then classifies transcripts from the original transcriptome assemblies into the following five categories:

- Clean (*Nf* > *XNa*)
- Cross contamination (*Nf* < *Na/X*)
- Dubious (*Na/X* < *Nf* < *XNa*)
- Over expressed (*N > Y* for at least three samples)
- Low coverage (*N* < Z in all samples)

### Analyses of six recent sequencing projects

**Fig. 1:**
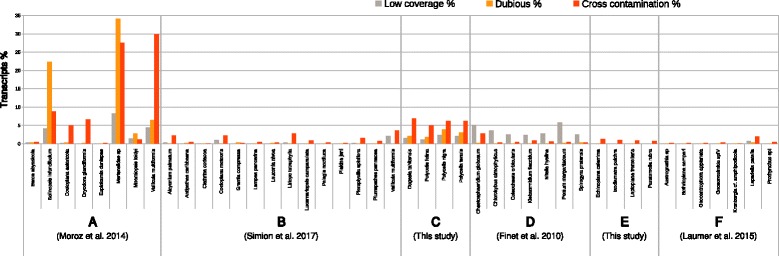
Pervasive cross contaminations observed in recent transcriptomic datasets from six different labs. For each transcriptome, three columns indicate the percentage of transcripts categorised as low coverage (*grey bars*), dubious (*orange bars*) and cross contamination (*red bars*) as detected by CroCo (using default parameters). For the content of each dataset, see Additional file 1: Table S1; references [16, 22, 34, 41]

We selected six datasets to be used as proof of concept for the cross contamination detection strategy implemented in CroCo (Additional file 1: Table S1). Note that the two datasets published in the context of the present study have been entirely processed by different authors in different labs; hence, they were analysed as two separate datasets. Transcriptomes from dataset C were assembled as described elsewhere [18]. Transcriptomes from dataset D were assembled using SOAPdenovo-Trans [19], and transcriptomes from dataset E were assembled using Trimmomatic [20] and Trinity v2.1.1 [21]. CroCo was used with default parameters (-f 2 -c 0.2 -d 300), and the results of these analyses are shown in Fig. 1.

### Impact of cross contamination on phylogenomics

**Fig. 2:**
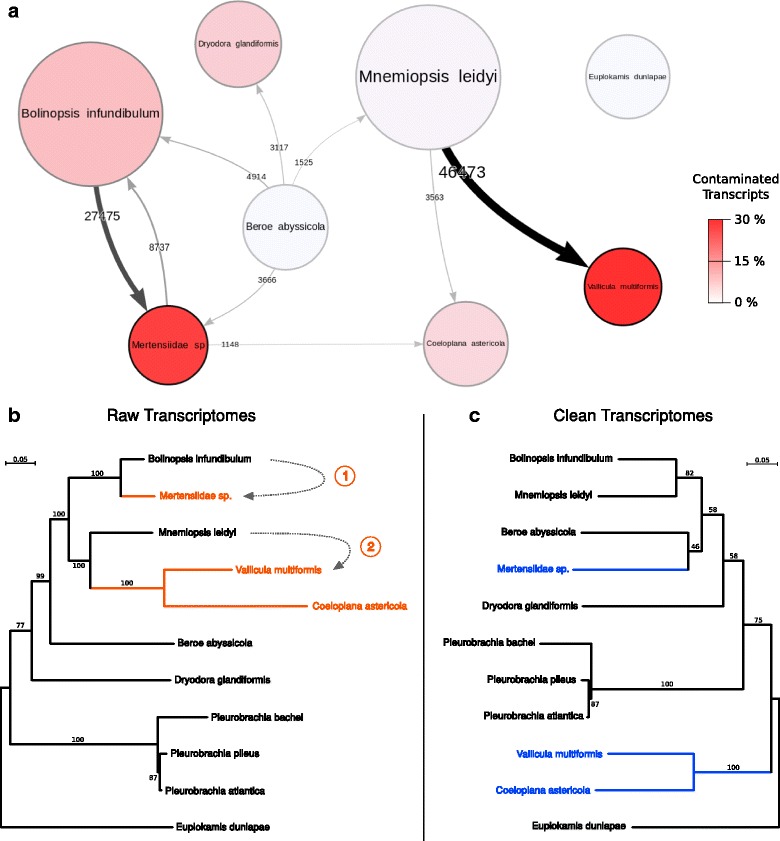
Dramatic effect of cross contaminations on reconstructing ctenophore relationships using a phylogenomic dataset. **a** Cross contamination network for dataset A as reconstructed by CroCo. Nodes and links represent transcriptomes and cross contaminations, respectively. Only transcripts strictly categorised as cross contaminants are taken into account here. Node sizes are proportional to the number of times the node is the source of cross contamination, and node colours represent the percentage of contaminated transcripts in the transcriptome. For clarity, weak links, defined as less than 2% of the strongest link in the network, are not shown. **b**, **c** Ctenophore phylogenetic relationships reconstructed with 114 genes using (**b**) untreated transcriptomes and (**c**) transcriptomes cleaned using CroCo (see details in Methods). In (**b**) the placement of lineages highlighted in *orange* disrupts the monophyly of the clade ‘Lobata’ (here represented by *Mnemiopsis leidyi* and *Bolinopsis infundibulum*). With the cleaned dataset (**c**), the same lineages, in *blue*, are placed in agreement with recent studies of ctenophore relationships [16, 31–33]. The two *dotted arrows* and their corresponding *numbers* indicate two major cross contamination events that can be observed on the cross contamination network (**a**)

In order to test the impact of cross contamination at a phylogenomic scale, we retrieved the 114 genes from a previous study [22], from which we kept only *Pleurobrachia* species sequences. To these 114 reduced alignments we added the raw ctenophore transcriptomic data from that study (i.e. dataset A, for which the cross contamination network is shown in Fig. 2a) using Forty-Two (available at https://bitbucket.org/dbaurain/42/). We then used SCaFoS [23] to concatenate the 114 completed alignments into a supermatrix, selecting the longest sequence if several sequences were present for a given species. We used RAxML [24] with the LG + Γ4 + F model of sequence evolution to infer phylogenetic relationships among ctenophores (Fig. 2b). We then used CroCo (using default parameters) to clean the raw transcriptomic data, and we retained all transcripts categorised as clean. We re-used the protocol described above to incorporate the cleaned data into the 114 genes, concatenated them and inferred a second cleaned phylogenetic tree of ctenophores (Fig. 2c).

### Example of cross contamination in a single-gene phylogeny

To exemplify the ability of CroCo to detect cross contaminations and their possible dramatic impact on gene phylogeny reconstruction, we arbitrarily selected a contaminated transcript from dataset A (i.e. transcript ‘sb|373879’ from the *Vallicula multiformis* transcriptome) and we used USEARCH [25] to extract homologous sequences from other transcriptomes from the same experiment (the parameters are as follows: --usearch_global --id 0.6 --maxhits 10000 --maxaccepts 10000 --maxrejects 10000). All sequences were then aligned with Clustal Omega [26] as implemented in SeaView [27] using default settings; the phylogenetic relationships between these transcripts were inferred by running 100 maximum likelihood searches under the LG + Γ4 + F model and 100 bootstraps with RAxML. Sequences were coloured according to their categorisation by CroCo (the parameters used were as follows: --tool B --fold-threshold 2 –minimum-coverage 0.2). The results are presented in Additional file 1: Figure S1.

### Cross contamination simulation experiments

#### Simulation of divergent transcriptomes

In order to test the accuracy of our procedure as well as to compare the behaviour of the different mapping/quantifying tools with closely related samples, we selected one reference transcriptome for subsequent simulations (*Austrognathia* sp. from dataset F; 31,529 transcripts). The abundance of each transcript was estimated in transcripts per million (TPM) using RSEM-Bowtie [28]. Based on this reference transcriptome, we simulated 10 divergent transcriptomes (of 31,529 transcripts each) using a Python script which randomly mutates nucleotides at a specified rate (divergence level ranging from 1 to 10%). The abundance of each divergent transcript is directly derived from the reference transcriptome. For each level of divergence, we worked on a pair of species including the reference and a divergent transcriptome.

#### Estimating effective contamination probabilities

To explain the cross contamination simulation procedure, we now focus on one such pair: the reference transcriptome and the 10% divergent transcriptome (hereafter ‘ref’ and ‘div10’, respectively). A given transcript originating from sample A which is transferred into sample B has a given probability to be effectively sequenced and assembled into a contaminating transcript in the transcriptome of sample B. For a given transcript, this probability should depend on the number of copies transferred (i.e. mRNAs) and thus on its abundance in sample A. These ‘effective contamination probabilities’ have been estimated by generating a virtual pool of one million mRNAs from the ‘div10’ transcriptome (based on the TPM of each transcript) in which we randomly sampled 10,000 mRNAs (corresponding to about 7300 unique transcripts). These transferred transcripts were added to the ‘ref’ transcriptome, and then 20 million reads were simulated from this contaminated transcriptome (as detailed in the following section). With a BLAST approach, we estimated how many of these transferred transcripts were recovered after transcriptome assembly with Trinity v2.4. The ‘effective contamination probabilities’ ranged from 48.3% (for the transcripts transferred in one copy) to 93.3% (more than 10 copies). Overall, about half of the transferred transcripts are recovered in the contaminated assembly.

#### Simulating cross contamination

These contamination probabilities were used to accurately simulate biologically realistic unidirectional contaminations from ‘div10’ to ‘ref’. Contamination was simulated by sampling 10,000 mRNAs from the pool of one million mRNAs of the ‘div10’ transcriptome. These 10,000 mRNAs correspond to N_1_ unique transcripts, of which only N_2_ (about 3600) will be effective contaminants based on their number of transferred copies and the corresponding contamination probability. The contaminated transcriptome thus comprises the 31,529 reference transcripts plus the N_2_ contaminating ‘div10’ transcripts. We performed the same procedure for a weaker contamination level, with an initial sampling of 1000 mRNAs. As we modelled contamination from one pool of one million mRNAs to another pool of one million mRNAs, each contaminating transcript was assigned a TPM corresponding to the number of copies in which it had been transferred. For each sample (‘ref’ contaminated and ‘div10’), 20 million paired-end reads were then simulated with the R package polyester [29] with a uniform error of 0.5% (following [30]). The number of simulated reads for each transcript was directly based on its TPM. For each divergence level (*n* = 10) and contamination strength (*n* = 2), CroCo was run with the three mapping tools (RapMap, Kallisto and Bowtie) using default parameters. The results are shown in Additional file 1: Figure S3.

#### Benchmarking CroCo with biological data

We categorised dataset E transcripts with CroCo (default settings) and then incorporated all transcripts into reference alignments maintained and updated by author HP and including taxonomic sampling corresponding to dataset E (see [13]), using the Forty-Two software package (https://bitbucket.org/dbaurain/42/downloads/). The taxonomic sampling already present in these alignments allowed us to determine the true origin of every transcript, which we compared to CroCo categorisation in order to evaluate its accuracy. The results of this comparison between manual and automated detection of cross contamination are shown in Additional file 1: Figure S2.

## Results

### Detecting cross contamination in six transcriptomic datasets

We used CroCo on six recent RNA-seq datasets, including two of our own, as a proof of concept (see details in Methods and Additional file 1: Table S1). These datasets correspond to the sampling of several different species spanning the diversity of a given group (e.g. Metazoa, Ctenophora, Platyhelminthes, Streptophyta). Within a given project, the mRNA of each species was sequenced by a single group of authors and hence potentially handled at the same place. We found that all of them were affected by cross contaminations and, more worryingly, that these cross contaminations can reach dramatic levels (i.e. almost 30% of all transcripts in one species, see Fig. 1). Sequencing experiments differ greatly in their levels of contamination (compare the highly cross contaminated datasets A and C with the cleaner datasets B and E in Fig. 1). The level of cross contamination also varies (sometimes greatly) between samples within each experiment, as is obvious when comparing *Euplokamis dunlapae* and Mertensiidae sp. in dataset A or *Lampea pancerina* and *Liriope tetraphylla* in dataset B (Fig. 1). This is consistent with some cross contamination stemming from experimental mishandling which is expected to result in preferential cross contamination patterns (i.e. only certain samples contaminate or are contaminated). These patterns can be easily observed on cross contamination network graphs rendered by CroCo which can be used to trace back the likely experimental step(s) at the origin of cross contamination event(s) (see Fig. 2a).

### Gene phylogeny in the presence of cross contamination

In order to illustrate both the problems caused by cross contaminations for phylogenetic studies and the ability of CroCo to detect them, we built a single-gene tree based on ctenophore (comb jellies) sequences from dataset A [22] prior to CroCo use. We then used CroCo to detect cross contaminations and correspondingly annotated the sequences in the tree (Additional file 1: Figure S1). Multiple cross contaminations clearly hampered the interpretation of the correct evolutionary history of this gene, but they were all detected by our procedure (i.e. none of them was categorised as clean). After their removal, we could reconstruct a gene phylogeny that is congruent with expected relationships between these ctenophore species (see Additional file 1: Figure S1 and [31–33]). This example shows the evident effect that undetected cross contamination can have when interpreting single-gene phylogenies.

### Evaluating the accuracy of CroCo

To further illustrate the accuracy of our approach, we used cross contaminations that have been previously identified as a reference [13]. Indeed, the potential negative impact of cross contaminations on phylogenomic inference has been studied using a previously published dataset [34] by comparing trees obtained before and after manual removal of contaminants. We thus evaluated CroCo’s ability to detect cross contaminants. Across all species, CroCo was able to detect 97.2% of the cross contaminations that have been identified manually (i.e. 174 out of 179 cases) while wrongly discarding < 1% of correct sequences (i.e. 6 out of 629 transcripts, see Additional file 1: Figure S2). We also evaluated CroCo’s accuracy by analysing simulated datasets with cross contaminations between transcriptomes that we made increasingly divergent from each other (from 1 to 10% divergence). We show that CroCo is able to detect 100% of cross contamination cases when samples display more than 2% average divergence in nucleotide sequences (Additional file 1: Figure S3). In addition, regardless of the choice of mapping strategy and sample divergence levels, less than 0.04% of clean transcripts were erroneously categorised as contaminant.

### CroCo as a cleaning tool for phylogenomics

As previously shown, cross contamination can be deleterious even at a phylogenomic scale where data quantity might be expected to be sufficient to overcome the erroneous signal from contaminants [13]. This happens because some cross contaminations are not random (see preferential cross contamination patterns in Fig. 2a and Additional file 1: Figures S4–S8) and can therefore create an additive non-phylogenetic signal that is reinforced as more and more genes are added (a case of systematic error). To assess the benefits of CroCo for phylogenomic analyses, we compared results from the supermatrix of 114 genes from the heavily contaminated ctenophore species of dataset A (Figs. 1 and 2a and Additional file 1: Figure S1) before and after cross contamination cleaning (see Methods). In the ctenophore tree reconstructed with raw transcriptomic data (Fig. 2b), the Lobata species *Bolinopsis infundibulum* is placed close to Mertensiidae sp., while the other representative of Lobata, *Mnemiopsis leidyi*, branches together with platyctenids (*Vallicula* and *Coeloplana*), both with maximal support. These relationships are at odds with ctenophore relationships previously derived from single-marker molecular phylogenies as well as with morphology [16, 31–33]. One of these problematic relationships has been recovered with maximal support in four recent studies based on dataset A [22, 35–37]. These two major incongruences match the two largest cross contamination events detected by CroCo in this dataset (events 1 and 2 marked in Fig. 2b, respectively corresponding to 27,475 and 46,473 cross contaminated transcripts; see black thick arrows in Fig. 2a). When we used only transcriptomic data categorised as clean by CroCo, we recovered a phylogeny congruent with the current understanding of ctenophore evolution (Fig. 2c), notably with the two Lobata species grouped together. This confirms cross contamination as a potential source of systematic error in phylogenomics.

## Discussion

### A quantitative tool to classify transcripts

CroCo has been designed to detect cross contamination in transcriptomes assembled from RNA-seq data of samples that were either multiplexed during sequencing or, more generally, handled by the same people or at the same location. Although we found evidence for systematic and pervasive cross contamination events, understanding CroCo’s strategy is important in order to interpret its categories correctly. Our approach is quantitative and relies solely on differential transcript quantification patterns to determine the origin of a given transcript. This allows CroCo to be reference-free and portable to any transcriptomic dataset, but it also renders its results sensitive to the accuracy of quantification estimates and to the user-defined parameters for categorisation.

The quality of both reads and transcriptome assemblies is important to maximise the accuracy of expression level estimates. For example, it is expected that transcriptome redundancy such as natural transcript variants might create false positive results, since they likely display different expression levels. Additionally, issues during transcriptome assembly might result in chimaeric transcripts that cannot be handled appropriately by CroCo. Data quality variation might explain most of the varying percentage of ‘low coverage’ transcripts in different datasets. The variation between dataset D (i.e. 454-based) to the others (i.e. Illumina-based) in that regard is certainly due to the sequencing technology used. Note that even considering that 454 pyrosequencing data are less adequate than Illumina data to quantify transcripts, this did not hamper CroCo from accurately detecting more than 97% of validated cross contamination cases. Overall, low coverage sequences usually correspond either to genuine transcripts with low expression or to bad quality mapping which may result from low-quality reads or inappropriate mapping strategies. On the other hand, transcripts categorised as ‘over expressed’ were found at low frequency in every transcriptome of every dataset. These transcripts typically correspond to ribosomal RNA sequences or common external contaminations, such as bacterial transcripts.

### CroCo parameterisation

Read mapping strategies and their implementation are an ongoing research area, leading us to implement three methods for transcript quantification in CroCo: Bowtie [38], Kallisto [39] and RapMap [40], which rely, respectively, on alignment, pseudo-alignment and quasi-mapping approaches. Using RapMap for transcript quantification yielded the most accurate results when analysing simulated cross contaminated datasets (Additional file 1: Figure S3). We thus set RapMap as the default quantification tool in CroCo, and advise the user that Bowtie should not be used for between-sample nucleotide divergence of less than 3%.

CroCo uses three parameters (see Implementation) to govern the categorisation of transcripts which can all be set by the user. If the ‘fold difference’ *X* parameter value is set low, CroCo will categorise more transcripts as either clean or as cross contaminated, whereas higher values will increase the number of dubious transcripts. The parameter *Y* is a quantification threshold above which a transcript is considered to be highly expressed. If a given transcript exceeds that threshold in three or more samples, which represents an unexpected and suspicious pattern, CroCo will categorise it as ‘over expressed’. Lastly, the parameter *Z* is the quantification level threshold under which a transcript is considered rare. A transcript found to be rare in every sample under study will be categorised as ‘low coverage’, under the rationale that our quantitative approach lacks power to confidently determine their true source dataset. Setting a combination of a high fold difference value, a low over expression threshold and a high low coverage threshold will result in high confidence cross contaminated and clean transcript assignments at the price of discarding a larger amount of data (see Additional file 1: Table S2). The CroCo user manual provides additional recommendations for setting these three values.

### Caveats when using CroCo

CroCo is designed to identify transcripts that conform to the expected profile of cross contaminations, but the user must use this information carefully. First, different decisions may be made on how to interpret or use the output depending on whether, for example, the user wants to avoid any possibility of cross contamination versus minimising the chance of discarding useful information.

There are three inherent limits to the approach implemented in CroCo. First, the expression level is expected to correlate with the quantity of input biological material. This implies that CroCo will not be able to detect cross contamination that occurred before any normalisation of the cDNA libraries under study. This also advocates for preferring sequencing data that allow more accurate estimates of transcript quantification (e.g. preferring Illumina data over 454 pyrosequencing).

A second obvious limitation is that if the species from which a given contamination originated is not included as input for CroCo, it will never be detected. Since a majority of cross contamination events can happen at a sequencing facility [9], we highlight the need for improved transparency in multiplexed taxon sampling from both researchers and sequencing service providers.

The third limitation is that the organisms under study must not be too closely related: the more closely related the samples are, the more difficult it will be to identify cross contamination. The method will fail when handling samples from individuals of a single species (e.g. comparing human samples). This limitation could be somewhat alleviated by setting more stringent values for parameters governing the BLAST step that determines the list of suspicious transcripts (see the user manual for additional recommendations). Even distinct species, if too closely related, share transcripts which, even if not identical, are likely to have similar numbers of matching reads. CroCo would place such transcripts in the ‘dubious’ category. If a true transcript in sample ‘A’ is expressed at a lower level than a very similar transcript in sample ‘B’, then it could theoretically be incorrectly classified as a cross contaminant. This outcome is most likely in cases when the sample size is small (less reliable expression quantification), when analysing two very closely related organisms (which is not recommended), and/or using an inaccurate mapping strategy. The higher amount of cross contamination observed in dataset C (Fig. 1) comprising closely related species might be a sign of over-estimation of cross contamination. Nevertheless, CroCo results on simulated cross contaminated datasets are reassuring regarding the capability of our tool for appropriately handling even closely related samples (Additional file 1: Figure S3), provided that their average genetic divergence is > 2%. Overall, the user needs to be aware of the limitations of the method in order to use it appropriately.

## Conclusions

Our results suggest cross contamination is a common issue in comparative molecular biology. Besides being detrimental for phylogenetic inference, it is clear that cross contamination can be massive enough to generate genome-scale impacts in comparative analysis of molecular data. It can also potentially adversely affect species delimitation, bias population genomic metrics, mimic other sources of incongruence between gene phylogenies (e.g. gene duplication, horizontal gene transfer, incomplete lineage sorting) or impact estimates of gene content. CroCo is a database-independent multi-platform tool that has been designed to be as easy to use as possible and that efficiently and accurately detects and removes cross contamination in assembled transcriptomes.

## Availability and requirements

**Project name:** CroCo

**Project home page:** http://gitlab.mbb.univ-montp2.fr/mbb/CroCo

**Operating system(s):** Platform independent

**Programming language:** Bash

**Other requirements:** R (optional); Docker (optional)

**License:** GNU GPL

**Any restrictions to use by non-academics:** none

## Additional file

Additional file 1: Figures S1–S8. Tables S1, S2. **Figure S1.** Single-gene phylogeny with multiple cross contaminations. **Figure S2.** Comparison between transcript categorisation by CroCo and a reference set of manually detected cross contaminations. **Figure S3.** Benchmarking CroCo using simulations. **Figure S4.** Network visualisation of cross contamination patterns in dataset B. **Figure S5.** Network visualisation of cross contamination patterns in dataset C. **Figure S6.** Network visualisation of cross contamination patterns in dataset D. **Figure S7.** Network visualisation of cross contamination patterns in dataset E. **Figure S8.** Network visualisation of cross contamination patterns in dataset F. **Table S1.** Datasets from six recent sequencing projects analysed with CroCo. **Table S2.** Effect of fold difference parameter value on transcript categorisations. (DOCX 1979 kb)
