# Supplementary material for: A software tool ‘CroCo’ detects pervasive cross-species contamination in next generation sequencing data
Source: BMC Biol. 2018 Mar 5;16:28. doi: 10.1186/s12915-018-0486-7 (PMC5838952; doi:10.1186/s12915-018-0486-7)

**Supplementary Figures and Table for “A software tool 'CroCo' detects pervasive cross-species contamination in next generation sequencing data”**

**Authors :**

Paul Simion^1,3^, Khalid Belkhir^1^, Clémentine François^1^, Julien Veyssier^1^, Jochen C. Rink^2^, Michaël Manuel^3^, Hervé Philippe^4,5^, Maximilian J. Telford^6^

**Affiliations :**

**1** Institut des Sciences de l'Evolution (ISEM), UMR 5554, CNRS, IRD, EPHE, Université de Montpellier, Montpellier, France

**2** Max Plank Institute of Molecular Cell Biology and Genetics, Pfotenhauerstrasse 108, 01307 Dresden, Germany

**3** Sorbonne Universités, UPMC Univ Paris 06, CNRS, Evolution Paris-Seine UMR7138, Institut de Biologie Paris-Seine, Case 05, 7 quai St Bernard, 75005 Paris, France

**4** Centre de Théorisation et de Modélisation de la Biodiversité, Station d'Ecologie Théorique et Expérimentale, UMR CNRS 5321, Moulis, 09200, France

**5** Département de Biochimie, Centre Robert-Cedergren, Université de Montréal, Montréal, H3C 3J7 Québec, Canada

**6** University College London, Centre for Life’s Origins and Evolution, Department of Genetics, Evolution and Environment, Darwin Building, Gower Street, London WC1E 6BT, UK


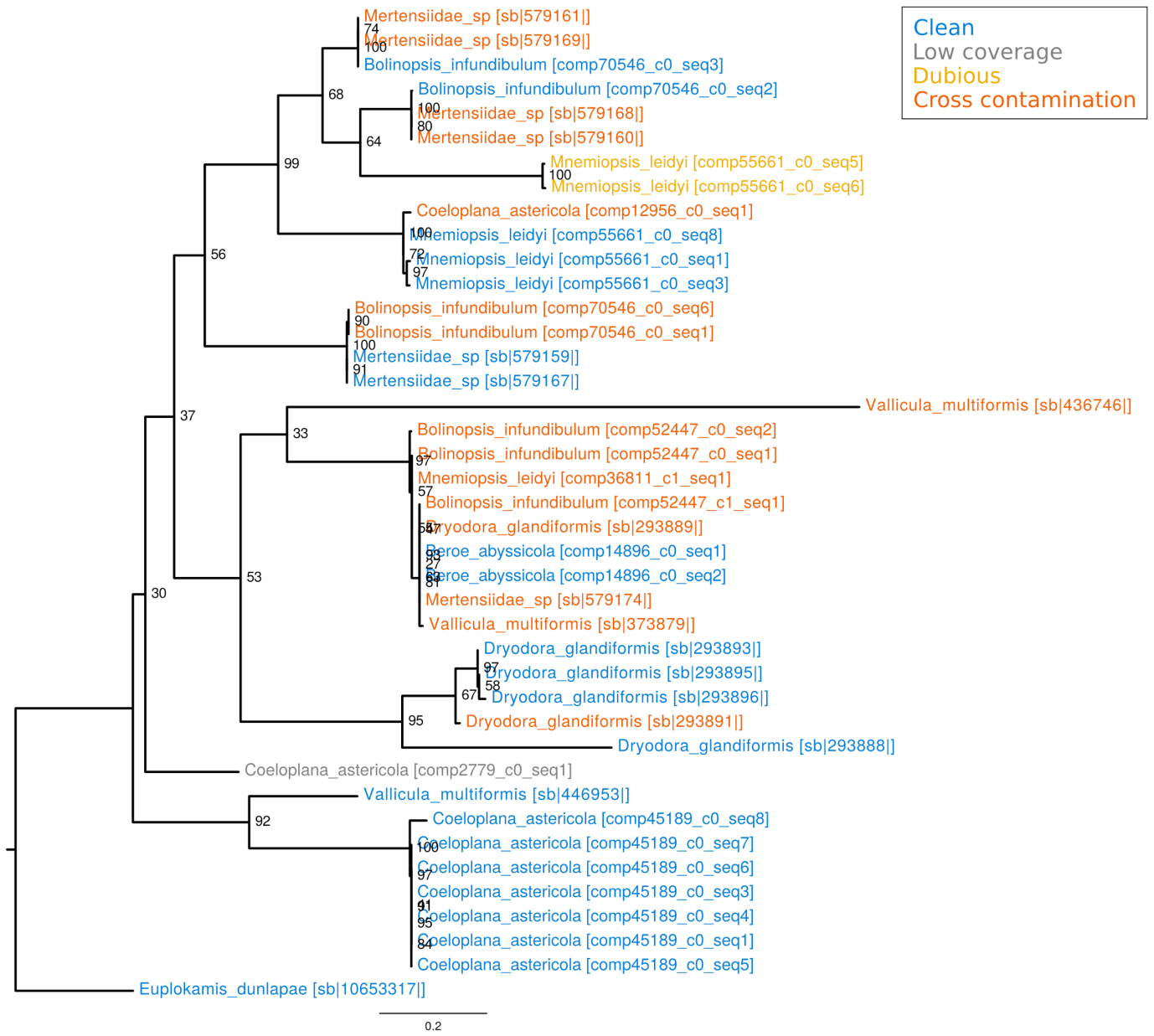


**Additional file 1: Figure S1:**

**Single-gene phylogeny with multiple cross contaminations**. Single-gene phylogeny reconstructed from a gene of dataset A belonging to the 14-3-3 gene family (see Methods section for details), showing at least 11 instances of cross contamination. We used CroCo to categorise transcripts and coloured them accordingly: blue for clean transcripts, grey for low coverage transcripts, orange for dubious transcripts and red for cross contaminations.


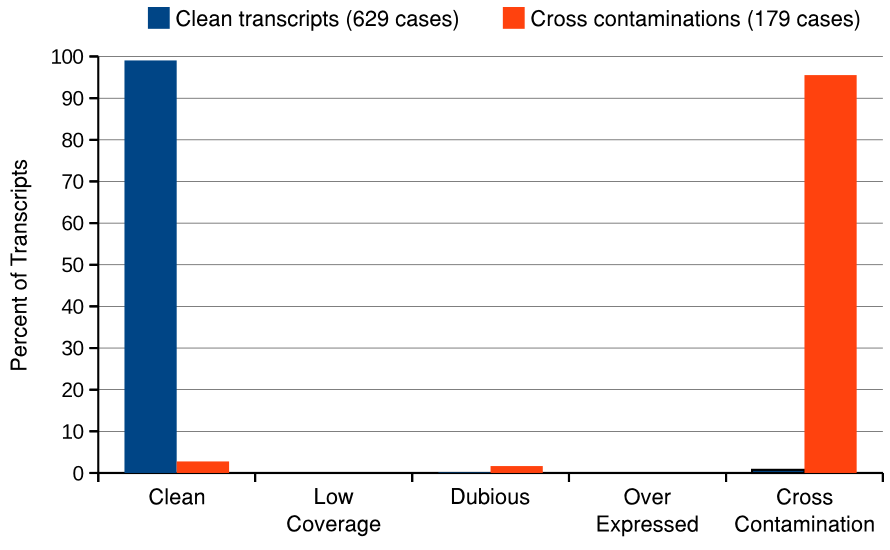


**Additional file 1: Figure S2:**

**Comparison between transcript categorization by CroCo and a reference set of manually detected cross contaminations.** CroCo categorization into five categories of transcripts previously classified as clean (629 cases, in blue) or as cross contaminations (179 cases, in red) using default parameters.

**
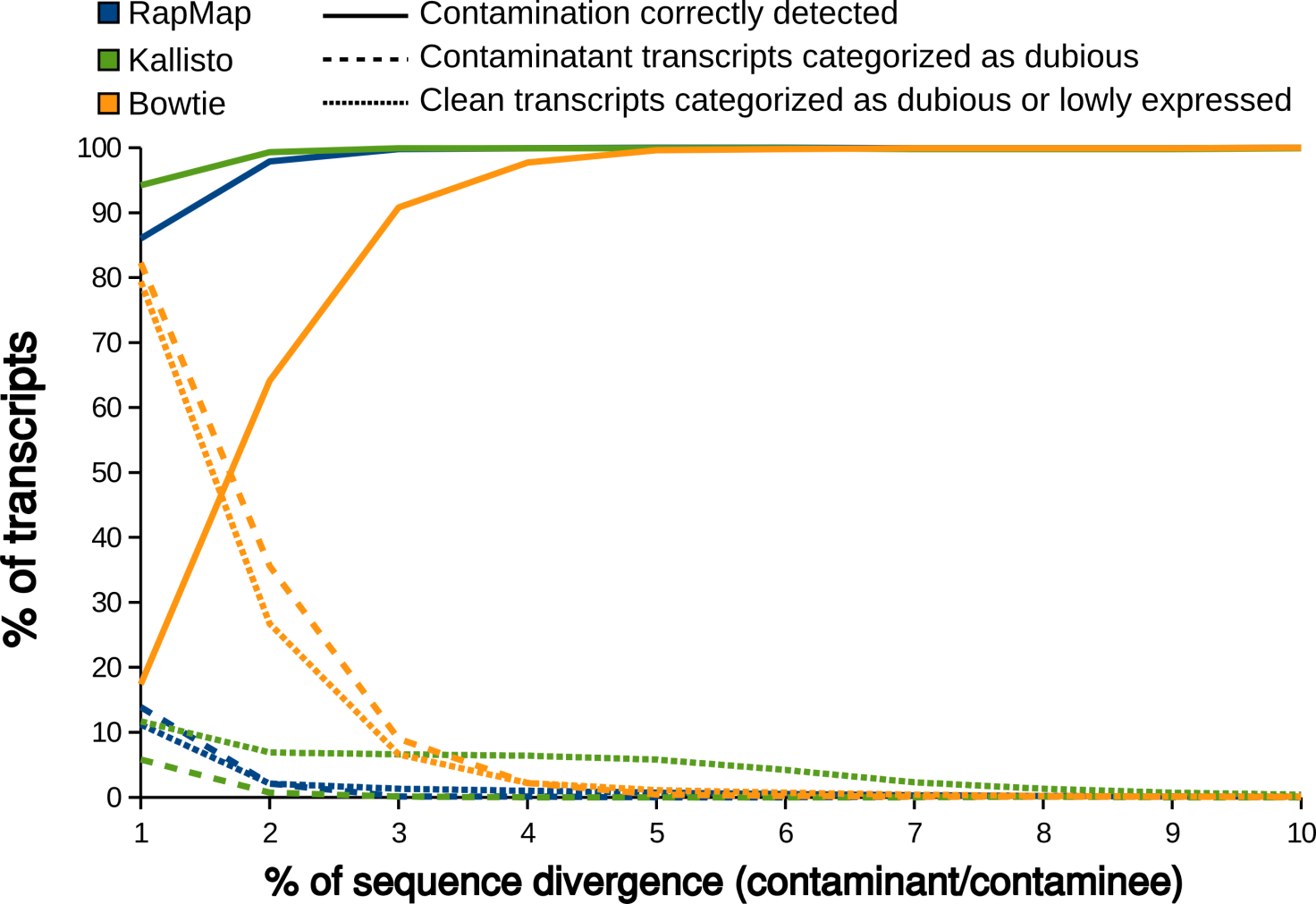
**

**Additional file 1: Figure S3:**

**Benchmarking CroCo using simulations.** Impact of genetic distance between the contaminant and the contaminee for three different mapping tools on the proportion of cross contamination correctly detected, cross contamination detected as dubious and clean transcripts categorized as anything other than clean. RapMap and Kallisto outperform Bowtie for this task.

**Note for network graph interpretation :**

Colors, nodes diameter and arrow sizes in networks are relative to the sampling used and therefore cannot be compared across different sequencing experiments. Example : although *Dryodora glandiformis* looks cleaner in fig. 2a than *Polycelis nigra* in Additional file 1: Figure S5 based on respective colors, both species have ~6% of their transcriptome that is contaminated.


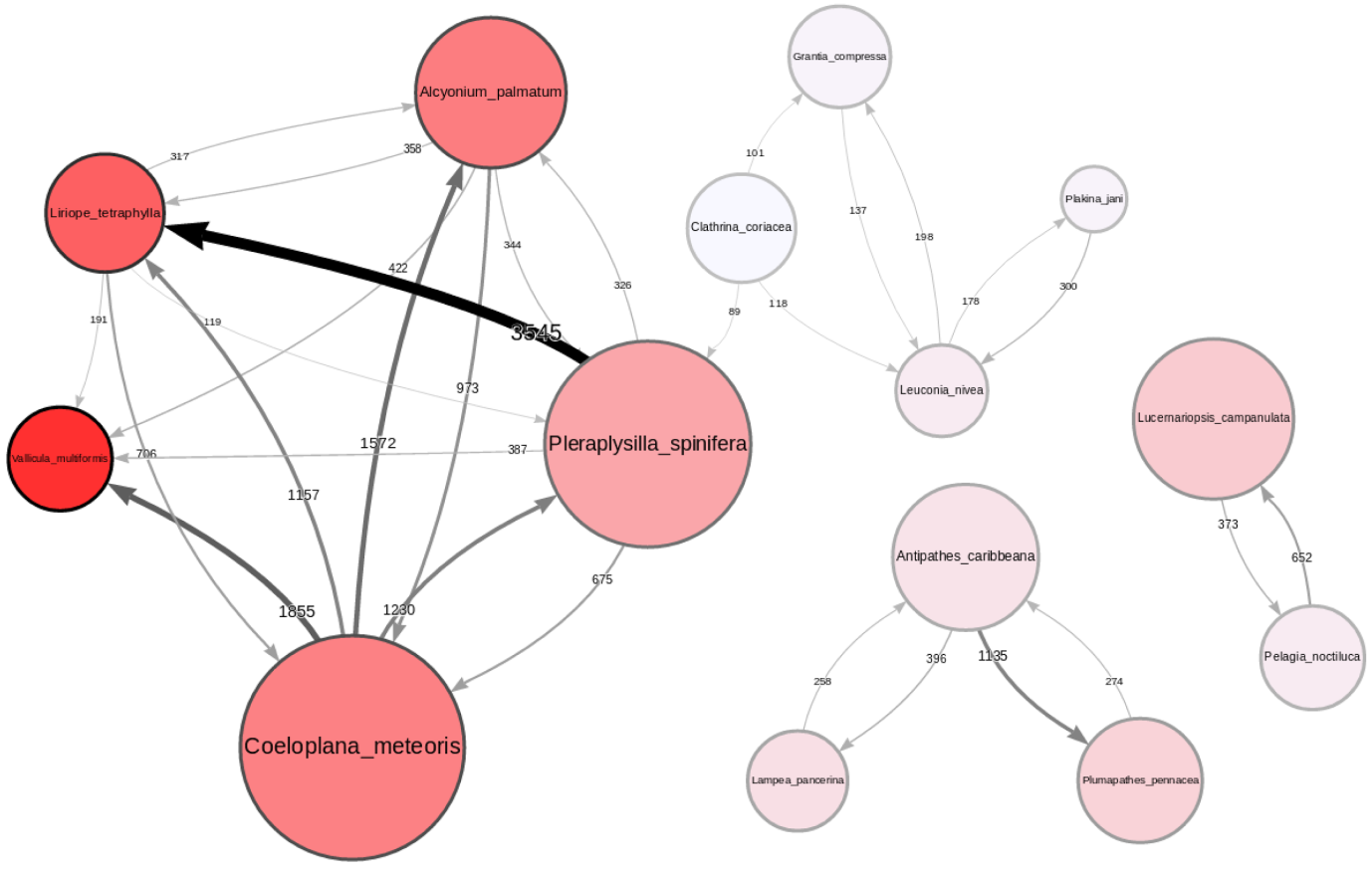


**Additional file 1: Figure S4:**

**Network visualisation of cross contamination patterns in dataset B**. Node diameter is proportional to the number of time the sample contaminates another one, node color represent the proportion of its sequence that are contaminated (from white to red), and arrow sizes represent the number of cross contaminations. For clarity, arrows representing less than 2% of the largest cross contamination link are not represented.


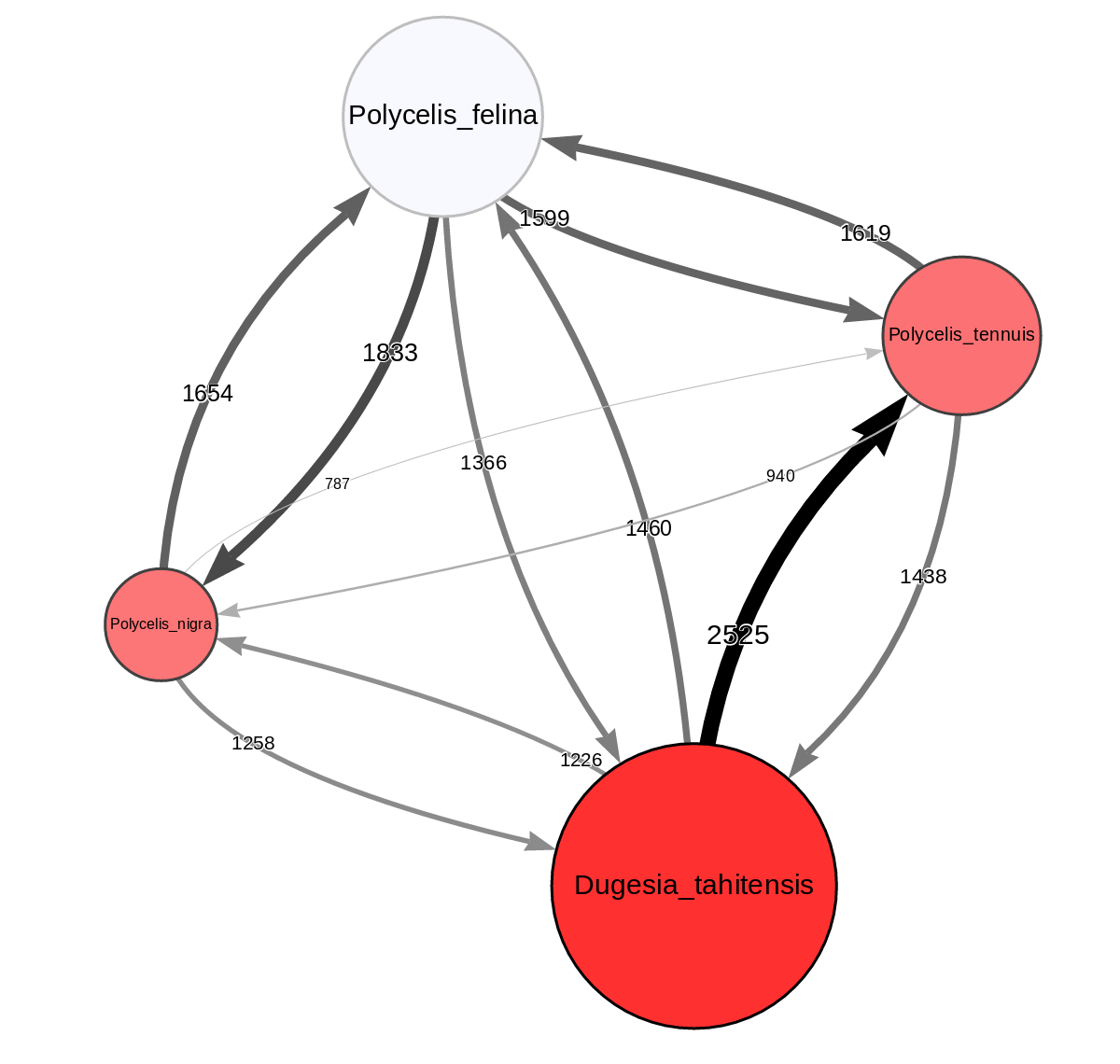


**Additional file 1: Figure S5:**

**Network visualisation of cross contamination patterns in dataset C**. Node diameter is proportional to the number of time the sample contaminate another one, node color represent the proportion of its sequence that are contaminated (from white to red), and arrow sizes represent the number of cross contaminations. For clarity, arrows representing less than 2% of the largest cross contamination link are not represented.


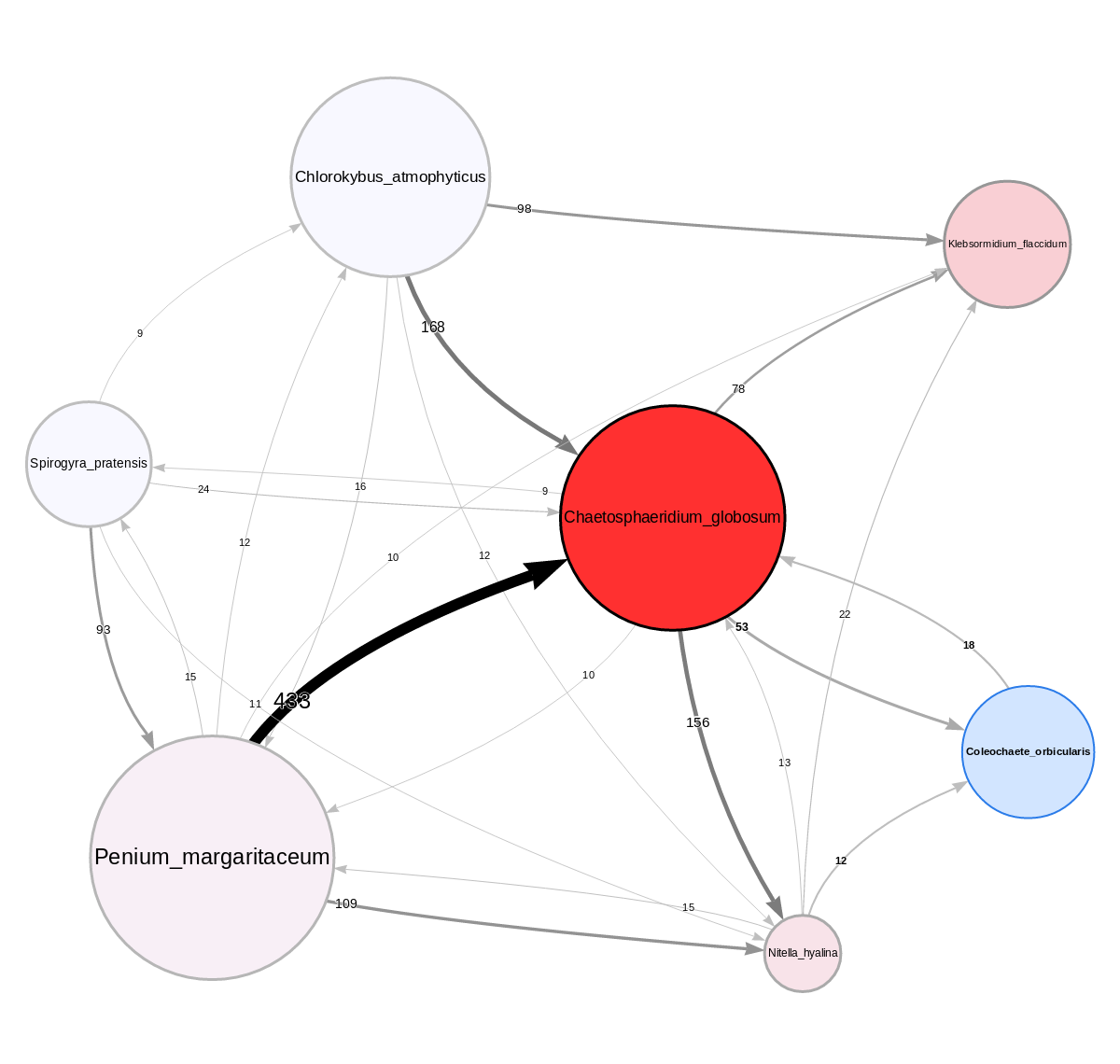


**Additional file 1: Figure S6:**

**Network visualisation of cross contamination patterns in dataset D**. Node diameter is proportional to the number of time the sample contaminate another one, node color represent the proportion of its sequence that are contaminated (from white to red), and arrow sizes represent the number of cross contaminations. For clarity, arrows representing less than 2% of the largest cross contamination link are not represented.


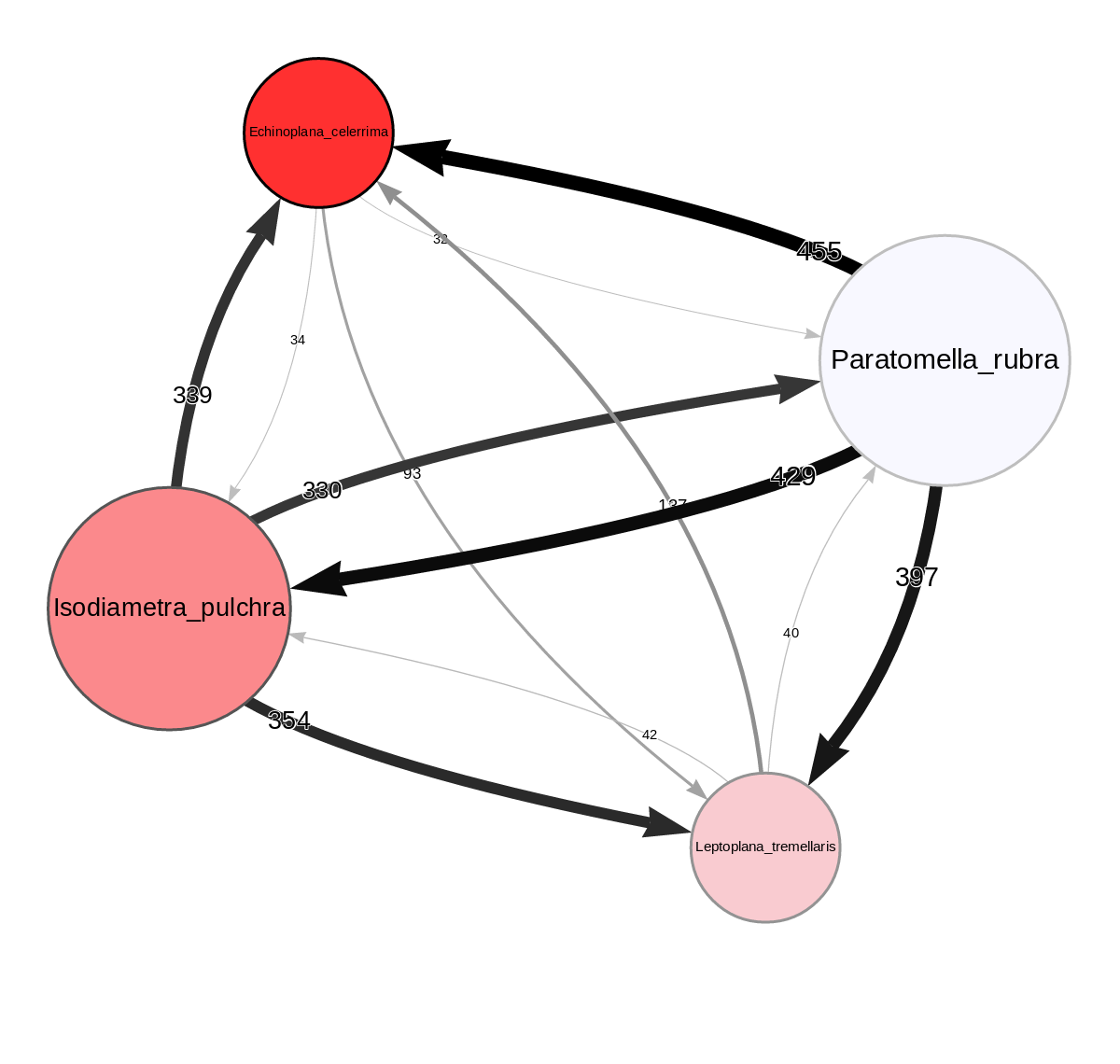


**Additional file 1: Figure S7:**

**Network visualisation of cross contamination patterns in dataset E**. Node diameter is proportional to the number of time the sample contaminate another one, node color represent the proportion of its sequence that are contaminated (from white to red), and arrow sizes represent the number of cross contaminations. For clarity, arrows representing less than 2% of the largest cross contamination link are not represented.


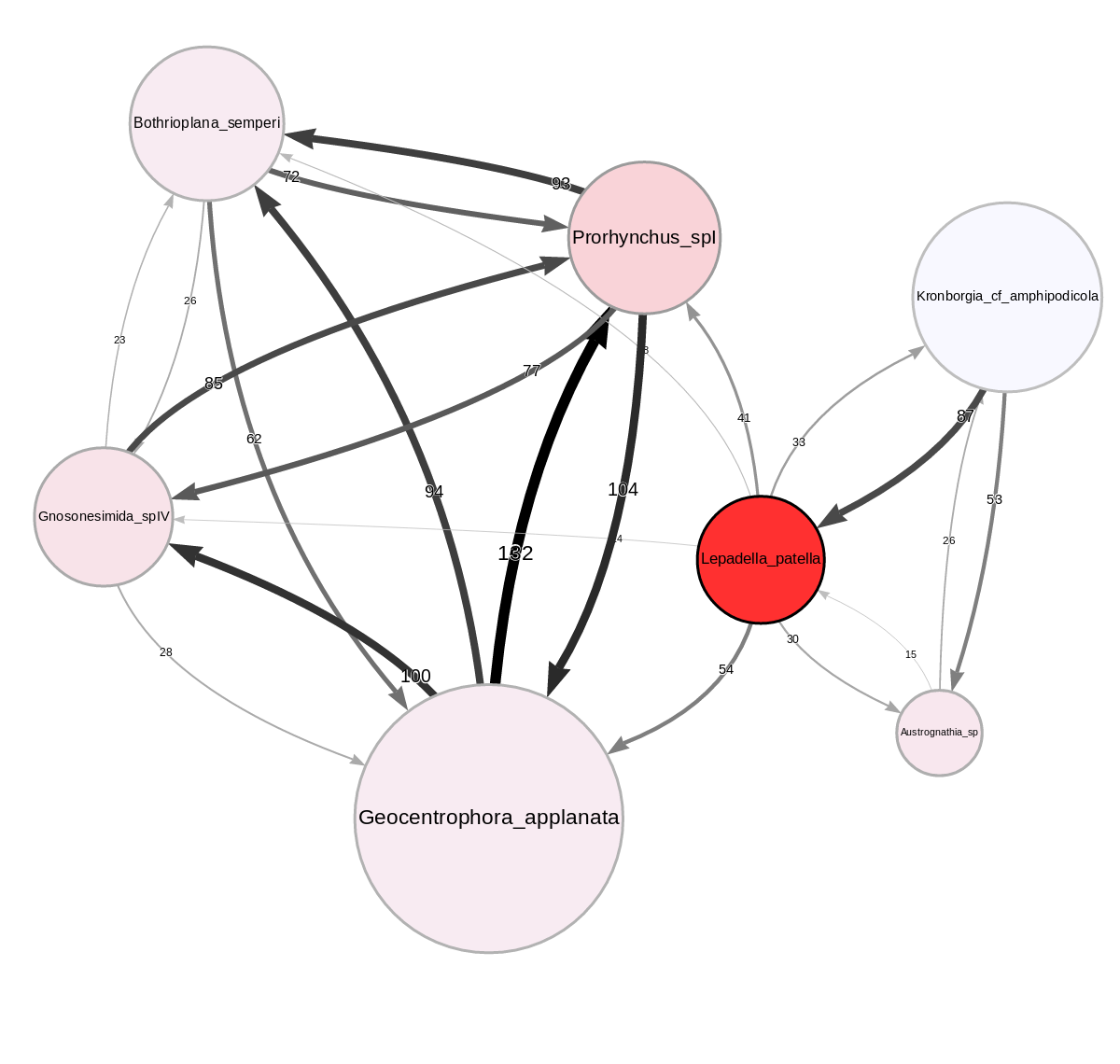


**Additional file 1: Figure S8:**

**Network visualisation of cross contamination patterns in dataset F**. Node diameter is proportional to the number of time the sample contaminate another one, node color represents the proportion of its sequence that are contaminated (from white to red), and arrow sizes represent the number of cross contaminations. For clarity, arrows representing less than 2% of the largest cross contamination link are not represented.

**Additional file 1: Table S1:**

**Datasets from six recent sequencing projects analysed with CroCo.** Datasets, species names, taxonomy and accession numbers for sequencing data.


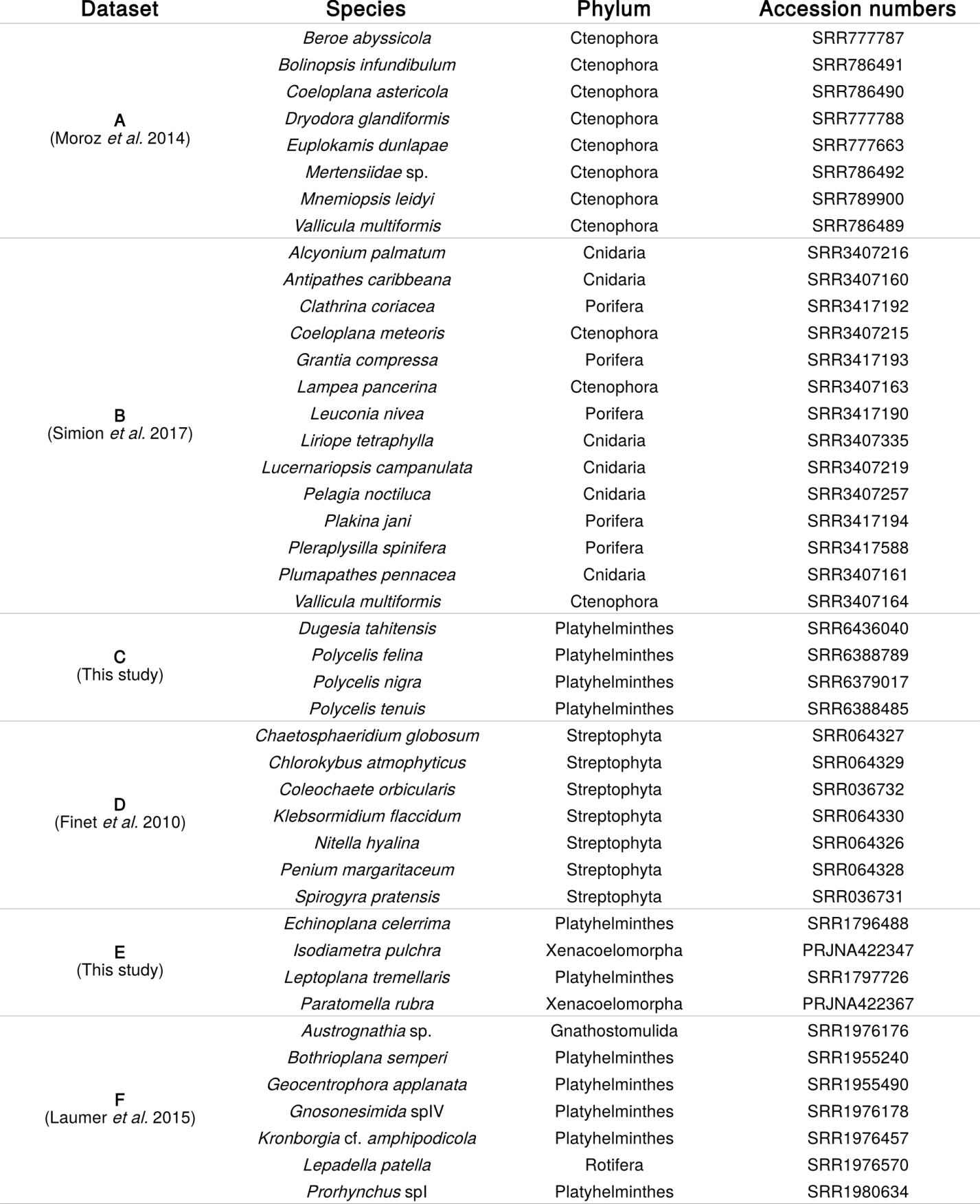


**Additional file 1: Table S2:**

**Effect of fold difference parameter value on transcripts categorizations.** Transcript catagories, value of the fold difference parameter, number of transcripts in *Mnemiopsis leidyi* and *Vallicula multiformis*.


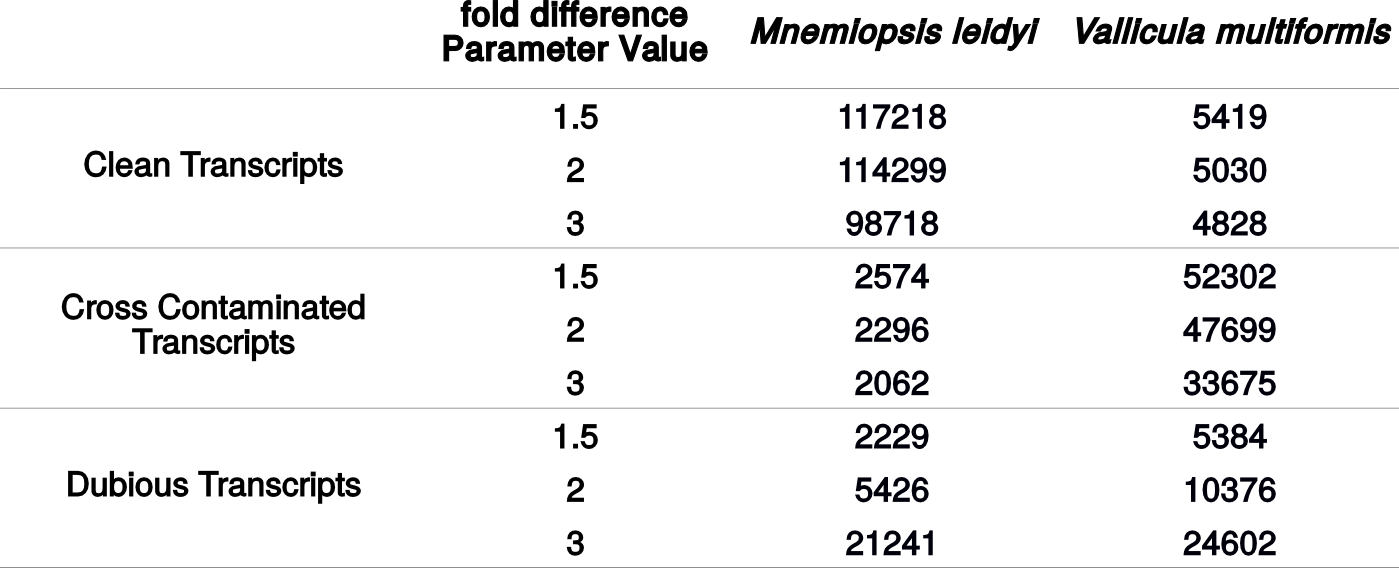

Supplement: Supplementary file 1 — Figure S1. Single-gene phylogeny with multiple cross contaminations. Figure S2. Comparison between transcript categorisation by CroCo and a reference set of manually detected cross contaminations. Figure S3. Benchmarking CroCo using simulations. Figure S4. Network visualisation of cross contamination patterns in dataset B. Figure S5. Network visualisation of cross contamination patterns in dataset C. Figure S6. Network visualisation of cross contamination patterns in dataset D. Figure S7. Network visualisation of cross contamination patterns in dataset E. Figure S8. Network visualisation of cross contamination patterns in dataset F. Table S1. Datasets from six recent sequencing projects analysed with CroCo. Table S2. Effect of fold difference parameter value on transcript categorisations. (DOCX 1979 kb) [file 12915_2018_486_MOESM1_ESM.docx]
